# Systematic Lab Knowledge Integration for Management of Lipid Excess in High-Risk Patients: Rationale and Design of the SKIM LEAN Project

**DOI:** 10.3389/fdata.2018.00004

**Published:** 2018-10-02

**Authors:** Chiara Pavanello, Marina Parolini, Antonia Alberti, Michele Carenini, Paolo Maino, Giuliana Mombelli, Franco Pazzucconi, Gianni Origgi, Federica Orsi, Maria Giovanna Trivella, Laura Calabresi, Renata De Maria

**Affiliations:** ^1^Dipartimento di Scienze Farmacologiche e Biomolecolari, Centro E. Grossi Paoletti, Università degli Studi di Milan, Milan, Italy; ^2^Dipartimento Cardiotoracovascolare, Istituto di Fisiologia Clinica del CNR, ASST Grande Ospedale Metropolitano Niguarda, Milan, Italy; ^3^SSD Diagnosi e Cure Territoriali per Malattie Cardiache, Dipartimento Cardiotoracovascolare, ASST Grande Ospedale Metropolitano Niguarda, Milan, Italy; ^4^Dedalus S.p.A., Bologna, Italy; ^5^Sistemi Informativi Aziendali, ASST Grande Ospedale Metropolitano Niguarda, Milan, Italy

**Keywords:** hypercholesterolemia, high cardiovascular risk, cardiovascular risk, LDL-cholesterol, electronic health records

## Abstract

SKIM LEAN aims at exploiting Electronic Health Records (EHRs) to integrate knowledge derived from routine laboratory tests with background analysis of clinical databases, for the identification and early referral to specialist care, where appropriate, of patients with hypercholesterolemia, who may be inadequately controlled according to their cardiovascular (CV) risk level. SKIM LEAN addresses gaps in care that may occur through the lack of coordination between primary and specialist care, incomplete adherence to clinical guidelines, or poor patient's compliance to the physician's prescriptions because of comorbidities or drug side effects. Key project objectives include: (1) improved health professionals' competence and patient empowerment through a two-tiered educational website for general practitioners (GPs) and patients, and (2) implementation of a hospital-community shared care pathway to increase the proportion of patients at high/very-high CV risk (Familial Hypercholesterolemia, previous CV events) who achieve target LDL cholesterol (LDL-C) levels. Thanks to a close collaboration between clinical and information technology partners, SKIM LEAN will fully exploit the value of big data deriving from EHRs, and filter such knowledge using clinically-derived algorithms to risk-stratify patients. Alerts for GPs will be generated with interpreted test results. GPs will be able to refer patients with uncontrolled LDL-C within the shared pathway to the lipid or secondary prevention outpatient clinics of NIG hospital. Metrics to verify the project achievements include web-site visits, the number of alerts generated, numbers of patients referred by GPs, the proportion of secondary prevention patients who achieve LDL-C <100 mg/dl or a >50% decrease from baseline.

## Introduction

Ischemic heart disease and stroke are the world's largest causes of death, accounting for a combined 15 million deaths in 2015^1^ Ischemic heart disease has been estimated to be the leading cause of disability in Europe, accounting for approximately 10% of total disability-adjusted life years. High plasma concentrations of low-density lipoprotein cholesterol (LDL-C) showed a strong linear relationship with the incidence of cardiovascular disease (CVD) (Castelli et al., 1992; Ference et al., 2017). Thus, as demonstrated by multiple randomized controlled trials, reduction of LDL-C is crucial for CVD prevention (Baigent et al., 2005, 2010). However European studies have highlighted extensive underdiagnosis and undertreatment of hypercholesterolaemia, particularly in some special populations, such as subjects with genetically-determined hypercholesterolemia (i.e., familial hypercholesterolemia), patients with known CVD (i.e., secondary prevention), diabetes or chronic kidney disease (CKD), as well as patients with high levels of individual risk factors (Nordestgaard et al., 2013; Reiner et al., 2013).

Familial hypercholesterolaemia (FH) is a monogenic autosomal dominant inherited disorder characterized by excessively high plasma levels of LDL-C from birth, due to a defect in the LDL receptor pathway. Without early detection and treatment, individuals with FH are at significantly elevated risk of premature ischemic heart disease due to accelerated onset of atherosclerosis. In the literature reported FH prevalence rates range from 1:200 to 1:500 (Hopkins, 2003; Nordestgaard et al., 2013; Schmidt et al., 2017). FH is relatively easy to detect and treat, but currently only an estimated 10% of FH cases are detected (Nordestgaard et al., 2013). Screening for FH through electronic health records has been suggested to represent the most systematic and cost-effective approach, with a high potential for integration into clinical practice. Studies in the primary care setting (Guglielmi et al., 2016; Safarova et al., 2016; Troeung et al., 2016; Vickery et al., 2017) have been conducted using automated detection algorithms based on laboratory lipid profile coupled to ICD coding and/or prescription of lipid lowering medications and Natural Language Processing for family history and presence of FH stigmata on physical examination, against a gold standard of modified Dutch Lipid Clinic Network diagnostic criteria. Estimates from Australia, Olmstead County in Minnesota US and Italy reported a prevalence of probable/definite FH ranging from 1:146 to 1:556.

Aref-Eshghi et al. applied a similar approach to the Canadian Primary Care Sentinel Surveillance Network database, and found that combining the history of medication therapy with abnormal lipid levels resulted in the best algorithm for the identification of subjects with dyslipidemia from electronic medical records, since the use of ICD diagnostic codes in primary care databases yielded a high number of false negative results. These authors did not however stratify their findings according to CV risk (Aref-Eshghi et al., 2017).

The need for a better control of cholesterol levels in Italy has been recently documented in an electronic registry in the primary care setting matched with administrative data on the use of medicines (Mazzaglia et al., 2009), in national studies (Poli et al., 2011, 2012) and international surveys (Reiner et al., 2013, 2016).

In a 2014 analysis of the national Health Search primary care database (Mazzaglia et al., 2009), the overall prevalence of dyslipidemia in the general practice setting was 15.8% (16.4% in women, 15.2% in men), increased with age and peaked at 34.1% between 66 and 75 years. Among subjects with diagnosed dyslipidemia, only 29.2% had LDL-C levels <100 mg/dl.

Statins represent an important share of drug expenses in Italy with a Defined Daily Dose of 67.6/1000 residents. Statins, the main cholesterol-lowering agents, are prescribed for primary prevention in 75% of cases, while only 56% of patients with a previous CV event or diabetes are on statins; however, overall treatment adherence rate is as low as 47%^2^.

The CHECK study analyzed CV risk as defined by the CUORE algorithm, derived by the National Health Institute from Italian cohorts^3^, in a primary care population of 5,456 subjects. CHECK documented a 24% prevalence of high-risk or very high-risk profiles (33% men, 16% women). Cholesterol levels beyond the desirable target were found in approximately one third of the overall cohort, 25% of diabetics and 26% of secondary prevention patients. Based on CHECK findings, almost 5 million Italians were estimated to need to reduce their LDL-C by over 37.5% (Poli et al., 2011). As for secondary prevention, the EuroAspire Surveys III (Reiner et al., 2013) and IV (Reiner et al., 2016) have documented in Italy LDL-C levels >100 mg/dl in 54.3% and 42.2% of patients with coronary heart disease, respectively.

Dyslipidemia guidelines recommend a staged approach based on risk stratification and the early identification and effective management of high-risk patients to tailor the intensity of LDL-C management (Catapano et al., 2016). In Milano metropolitan area, which serves approximately 1,400,000 residents, CV disease was in 2013 the first cause of death in women (34.4%) and the second in men (29.5%) and a leading cause of hospital admission (standardized rates 25/1,000 in men and 15/1,000 in women). From the Local Health Authority database, proportions of Milano residents with overt CV disease or diabetes were 10.4%, 3.6% among 40–64 years old subjects and 38% and 11% among those >65 years, respectively. Of local residents with laboratory tests performed, 28% self-reported hypercholesterolemia; of these 24% were on drug therapy as compared to the 29% national Italian rate.

ASST Great Metropolitan Hospital Niguarda (NIG) is a tertiary referral center with wide outreach. The catchment area of its biochemistry laboratory is largely coincident with Milano Local Health Authority District 2^4^, with a population of 350,000 residents. Over 2,000 patients were admitted for a CV event or procedure at NIG in 2014. Based on the above data, we estimated that over 50,000 patients in NIG catchment area might benefit from cholesterol-lowering agents, whereas in the primary prevention setting, given estimated prevalence rates of 1:200 to 1:500 of familial hypercholesterolemia in the literature, 700–1.750 FH subjects may live in NIG catchment area.

SKIM LEAN was devised to exploit the potential of electronic health records (EHRs) stored at a large metropolitan hospital. The general objective of SKIM LEAN is to implement the systematic integration of knowledge derived from routine laboratory tests with background analysis of EHR clinical databases for the identification and appropriate early referral to specialist care of patients with hypercholesterolemia, who may be inadequately controlled according to their CV risk level. SKIM LEAN will risk-stratify subjects undergoing lipid testing at biochemistry laboratory, to alert their GPs for abnormal values, and to offer specialist advice on lipid management if appropriate.

Furthermore, SKIM LEAN aims at improving health professionals' competence and patient empowerment, as well as implementing a hospital-community shared care pathway to increase the rate of patients who achieve LDL-C targets.

SKIM LEAN is composed of a retrospective epidemiological analysis phase and a prospective shared care pathway interventional phase.

The aim of the first retrospective phase was to stratify patients in CV risk categories, to identify subjects with possible FH or patients with a previous atherosclerotic CV event (secondary prevention) (i.e., at very-high CV risk), and to assess the discrepancy between ideal targets and the current concentration of LDL-C observed in these subgroups. This paper describes the strategies and methods used for the epidemiological analysis and the implementation of the shared care pathway, and reports the results obtained from data extracted during 2016.

## Materials and methods

All male and female subjects aged ≥ 18 years with laboratory testing performed at NIG starting from 1/1/2016 were retrospectively enrolled in the study.

The Ethical Committee Milano Area 3 of the ASST Grande Ospedale Metropolitano Niguarda approved the retrospective analysis of data extracted from hospital database, and waived the need for further consent to be obtained according to national data protection regulations (Authorisation no. 9/2014—General Authorisation to Process Personal Data for Scientific Research Purposes Published in Italy's Official Journal No. 301 of 30 December 2014^5^; written informed consent will be obtained from all research participants when they attend the clinic for prospective enrollment.

**Figure 1:**
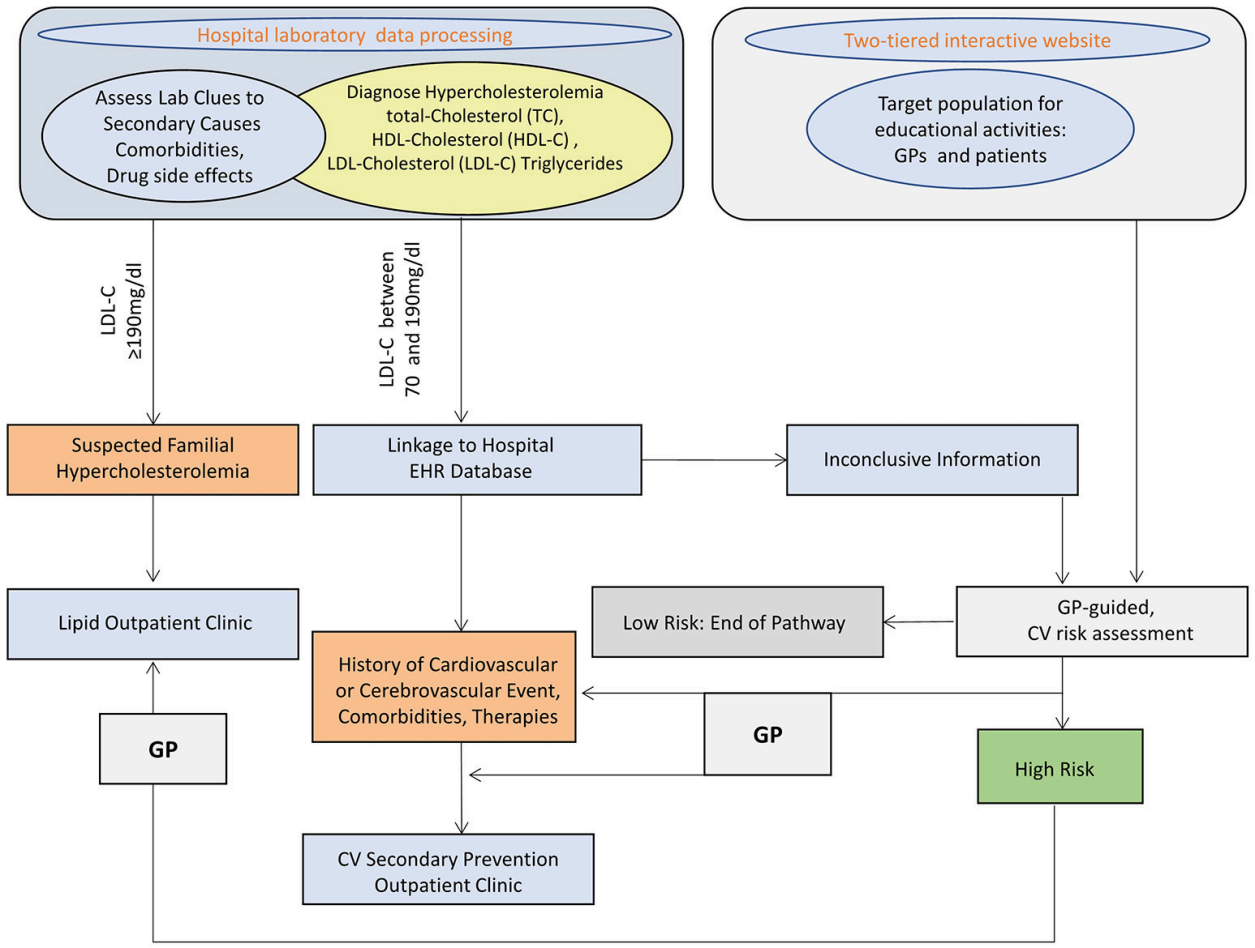
SKIM-LEAN project design. TC, total cholesterol; HDL-C, high density lipoprotein-cholesterol; LDL-C, low density lipoprotein-cholesterol; EHR, electronic health records; CV, cardiovascular; GP, general practitioner.

Study design is summarized in Figure 1.

The digital tool developed for the project has been integrated into NIG Information System. All study information flows occur through the web enterprise-wide intranet NIG Clinical Portal. The Portal complies with requirements by local regulations and Joint Commission standards as far as data security, traceability, and user identification are concerned.

Production databases includes laboratory test results from the Laboratory Information System (LIS), EHR and admission-discharge transfer (ADT). All extracted data of interest are conveyed to the engine containing rules and correlation algorithms that may be triggered through a user interface accessible by the different actors involved.

Per each subject, data extraction starts from the LIS, which collects and processes data from laboratory tests performed at NIG. The LIS scans past examinations and, through a batch Extract Transfer Load (ETL) procedure, drop selected datasets on cases in an interchange database.

LIS data are integrated with selected clinical databases coming from the EHR and ADT in NIG Clinical Portal to assess whether patients had previous CV events, clinical modifiers for high CV risk (diabetes, CKD, hypertension), or other comorbidity potential secondary causes of hypercholesterolemia or conditions that may pose a possible risk of adverse drug events, and in addition to verify prescribed therapies.

Automated algorithms were built to extract such cases of interest. The algorithm classifies subjects according to coded diagnosis procedures and pharmacological therapy recorded in the individual EHR within the previous 5 years (2011 to 2015). For those subjects who had no previous coded diagnosis registered in the Clinical Portal, working diagnosis reported on outpatient test prescription forms (mandatory for Italian regulations) were used. Text mining was based on Italian language terms, considering possible misspelled term combinations.

When neither coded nor working diagnosis were available, classification was attributed on the basis of the hospital unit were blood samples were drawn.

Selection rules and coding algorithms for subjects classification are shown in Table 1.

**Table 1:** Selection rules and coding algorithms to classify risk level of examined subjects.

***1. Secondary Prevention (previous CV event)***	**1.1–Ischemic heart disease (IHD)**		ICD9CM code 410.xx to 414.xx. Working diagnosis text mining terms “INFART,” “CAD,” “IMA,” “ISCHEM,” “ANGI,” “CORON,” “PTCA”
	**1.2–Cerebrovascular disease (CVD)**		ICD9CM code 433.xx to 437.xx. Working diagnosis text mining terms ICTUS,“ “CAROT,” “CEREB,” “ICA,” “TSA,” “TIA”
	**1.3–Peripheral vessel disease (PVD)**		ICD9CM code 440.2x Working diagnosis text mining terms “ANEURIS,” “AOCP,” “ARTERIOP,” “VASCULOP,” “CLAUDICATIO,” “ATERO”
***2. Excluded for potentially secondary hypercholesterolemia***	**2.1–HIV**		ICD9CM code 042, V08, 79571, 07953. Working diagnosis text mining terms “HIV,” “IMMUNODE,” “ART,” “ACAVIR”
	**2.2–Hypothyroidism**		ICD9CM code 243, 244.xx, 245.xx. Working diagnosis text mining terms “IPOTIR“
	**2.3–Epilepsy**		ICD9CM code 345.xx. Working diagnosis text mining terms “EPIL”
	**2.4–Nephrotic syndrome**		ICD9CM code 581.xx
	**2.5–Organ transplant**		ICD9CM code V42, V420, V421, V426, V427, V428, V4281, V429. Working diagnosis text mining terms “TX,” “TRAP,” “OLT”
	**2.6–Previous procedure** as exclusion criterion		ICD9CM code 312, 335.x, 336, 410.x
	**Drug therapy** as exclusion criterion		ATC D10BA01, H02, J05, N03
***3. High risk***	**Established**	**3.1-Diabetes**	ICD9CM code 250.xx. Working diagnosis text mining terms “DIAB,” “DM,” “GLICEM,” “GLUC,” “MELLI,” “13”
		**3.2-Chronic kidney disease (CKD)**	ICD9CM code 580.xx, 582.xx, 583.xx, 585.xx, 586, 587. Working diagnosis text mining terms “IRC,” “INSUF REN,” “DIALI,” “ED,” “NEFRIT,” “GMN”
		**3.3 -Hypertension**	ICD9CM code 401.xx to 405.xx. Working diagnosis text mining terms “IPERT”, “IPA”, “IPART”, “PRESSOR”, “IA”
	Putative	**3.4-Dyslipidemia and Obesity**	ICD9CM code 272.xx, 278.xx. Working diagnosis text mining terms “DISL,” “DISPIL,” “DISPLI,” “IPERCO,” “IPERLI,” “IPERTRI,” “COLEST,” “OBES,” “S METABOL,”“DISMETAB,” “TRIGLI,” “LIPID,” “MALNUT ECCES,” “25”
		**3.5–Rheumatic diseases**	ICD9CM code 710.xx, 714.1, 714.2, 714.3x, 7200, 725. Working diagnosis text mining terms “ARTRITE REUM,” “BECHET,” “BEHCET,” “SJOGREN,” “STRAUS,” “CONNET,” “SCLERODERM,” “SCLERO SISTEM,” “AR,” “LUPUS”
		**3.6–Chronic hepatitis cirrhosis**	ICD9CM code 571.4x, 571.5, 571.6, 571.8, 571.9. Working diagnosis text mining terms “EPATO CRON,” “EPATIT,” “HCV,” “HBV,” “NASH,” “ECA,” “STEAT,” “CIRROSI,” “NAFLD,” “16”

Subjects who according to the selection algorithms shown in Table 1 did not fit in any risk category s or blood donors (identified by the hospital unit “Immunohematology and transfusion medicine” code) were classified as “primary prevention patients at low CV risk.”

Patients with more than one diagnosis were classified in decreasing order of severity: e.g., a hypertensive subject who had a history of CV event was categorized as secondary prevention.

Data iteratively extracted from the different sources are merged on the Clinical Portal study pages for individual cases, in order to allow subsequent processing for referral to the shared care pathway. Data for patients with LDL-C >70 and <190 mg/dL and high CV risk and all those with LDL-C ≥190 mg/dL were considered eligible for selection to enter the prospective study. For this specific activity, the algorithm allowed to drop subjects with LDL-C already at target.

For all eligible subjects a patient page is made available on the Clinical Portal dedicated section to allow review by clinical investigators and confirmation of status.

On this page clinical investigators can:

◦ Access a case profile sheet merging key data from the above-mentioned sources
◦ Navigate through past patients' records stored on the Clinical Portal EHRs
◦ Navigate through past patients' medical reports through the hospital clinical repository
◦ Delete from the lists cases classified as “not interesting” by the selection algorithm
◦ Validate high-risk cases proposed by the algorithm for alerting the GP
◦ Classify relevant cases for further investigation by the GP
◦ Fill-in administrative patient data (as, e.g., GP contacts) if the system detects missing information.

Alerts for GPs are generated in a standardized report format for those patients who are judged to have inappropriately high LDL-C for their CV risk level, and might potentially need a specialist referral to NIG outpatient secondary prevention clinic or lipid clinic.

The report is posted through the Regional Health System Repository in patients' individual EHR and an alert email for GPs is automatically generated. Two links are embedded in the report. The former points to the hospital web-page detailing SKIM LEAN project objectives and processes, so that GPs can always refresh their knowledge on SKIM LEAM and decide whether they consider specialist referral appropriate for the specific patient. The latter link allows GPs to register the patient's consent and willingness to access the prospective shared-care pathway. GPs responses update the patients' status on the SKIM LEAN page in the Clinical Portal, where a list is generated for visit booking by personnel at the hospital clinics.

## Evaluation of practice improvement objectives

Systematic and repetitive processing of NIG laboratory test results during project year 2 will allow to assess changes in rates of uncontrolled hypercholesterolemia at the population level for all the subjects who undergo LDL-C testing, and treatment and patient-level changes in LDL-C targets for those patients who enter the care pathway.

Specific metrics for practice improvement objectives are:

- Proportion of secondary prevention patients who achieve LDL-C < 100 mg/dl or a >50% decrease in LDL-C (primary outcome end point). We calculated that, based on national and literature data (Poli et al., 2011) and an estimated drop-out rate of 40% (non-referral by GP, or patient refusal, or patient decline to repeat follow-up LDL-C), 550 secondary prevention patients should be selected to detect an absolute 10% points increase (from 50 to 60%) in the above metric with 80% power and alpha 0.05.
- Increase in the overall proportion of patients with LDL-C <100 mg/dl from baseline to end of study data extraction (secondary end point): 1500 patients with follow-up LDL-C tests will allow to detect an absolute 5% points increase (from 40 to 45%) in this metric with 80% power and alpha 0.05.
- Absolute/percent change in individual cholesterol levels for all patients who have follow-up lab tests from the first to the second study year.

### Retrospective epidemiological analysis of 2016 data

**Figure 2:**
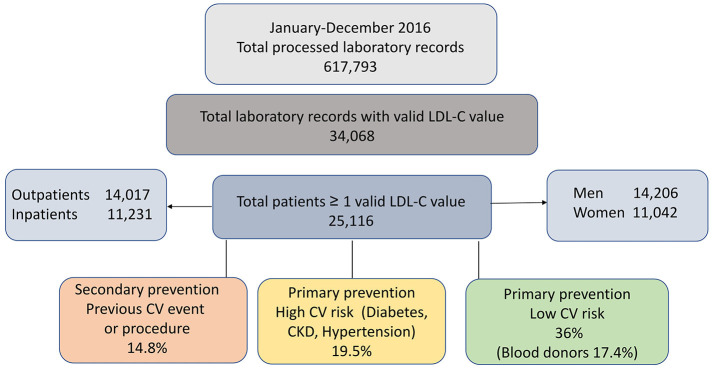
SKIM-LEAN data flow for the period January–December 2016. CKD, chronic kidney disease.

Based on the detailed criteria described in the method section, data from 25,116 patients with tests charged to the Regional Health service during 2016 and at least one valid LDL-C result available were identified. The enrolment flow is depicted in Figure 2.

**Figure 3:**
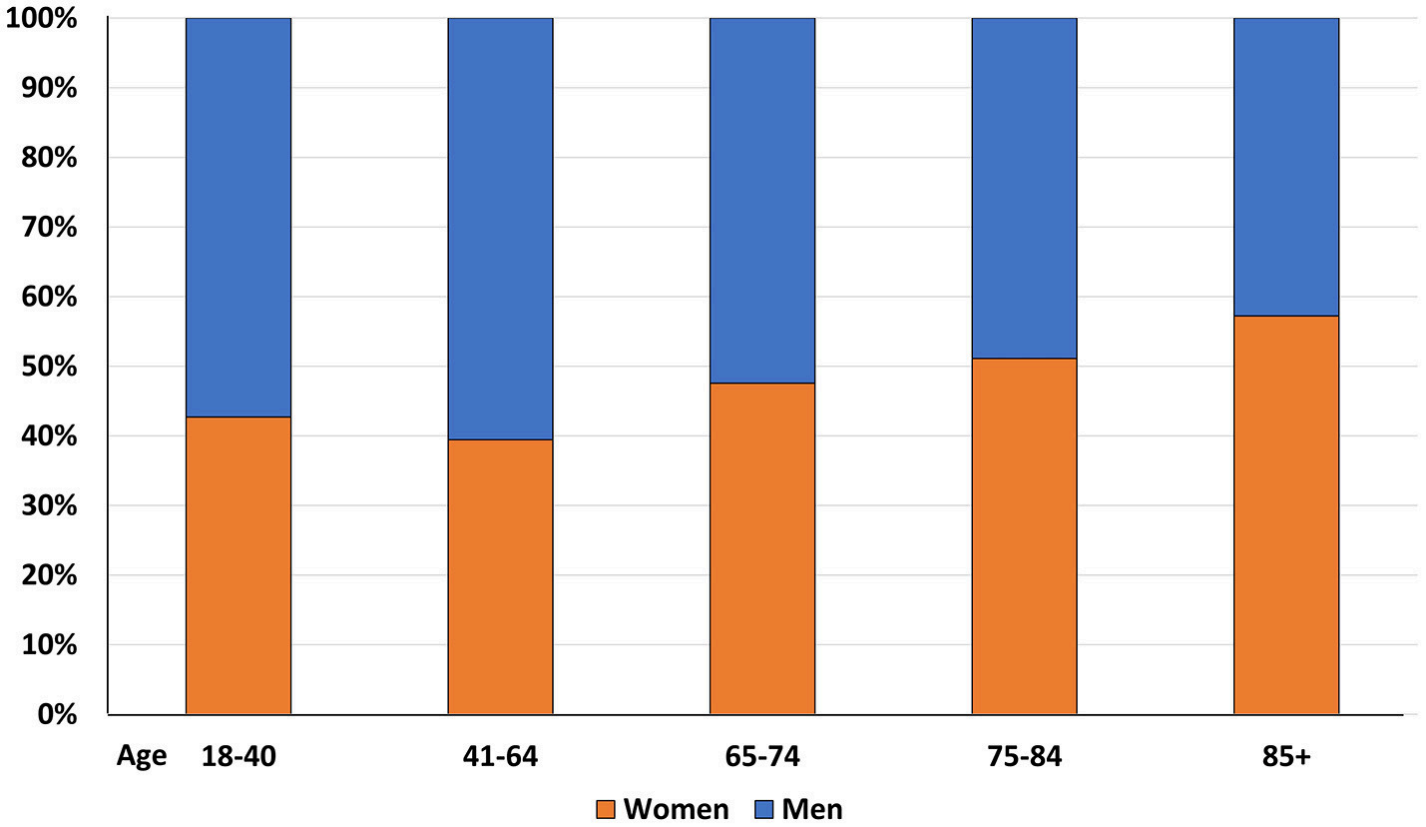
Gender distribution by age categories.

Consistently with well-known demographic trends, the proportion of female subjects significantly increased with age (*p* < 0.0001) (Figure 3)

**Figure 4:**
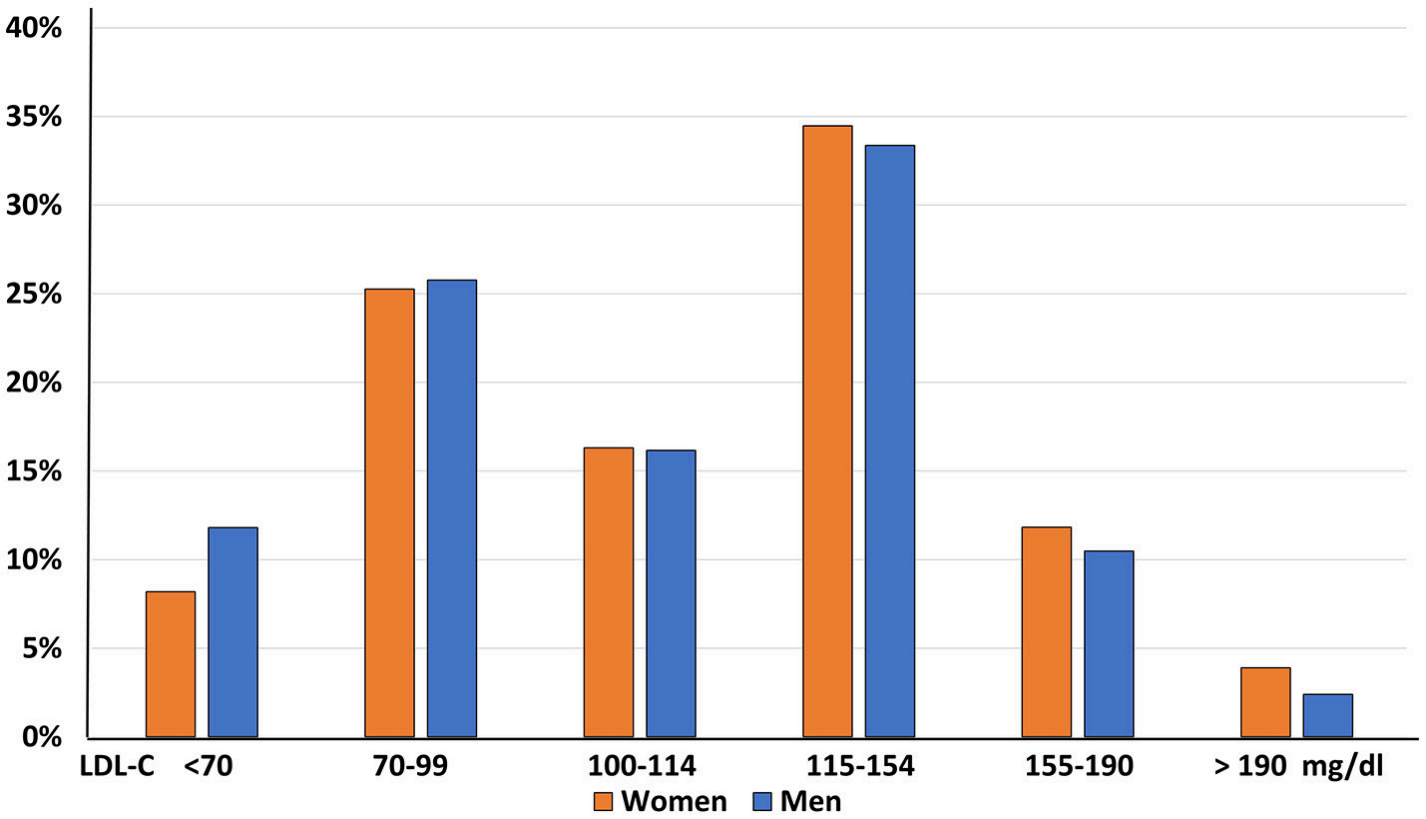
LDL-C distribution by gender.

Furthermore, a significant imbalance in LDL-C distribution across genders was observed, with a significantly greater prevalence of women in the higher LDL-C classes (*p* < 0.0001, Figure 4).

**Figure 5:**
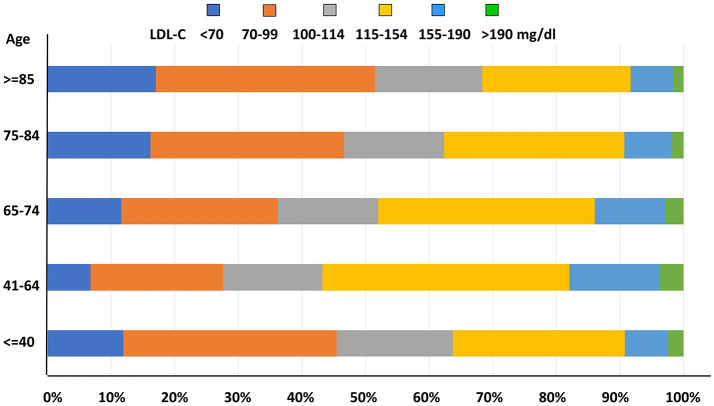
LDL-C distribution by age categories.

As for LDL-C level distribution with age, prevalence of uncontrolled LDL-C concentrations was significantly higher among middle-aged subjects when compared to younger and older patients (*p* < 0.0001, Figure 5).

**Figure 6:**
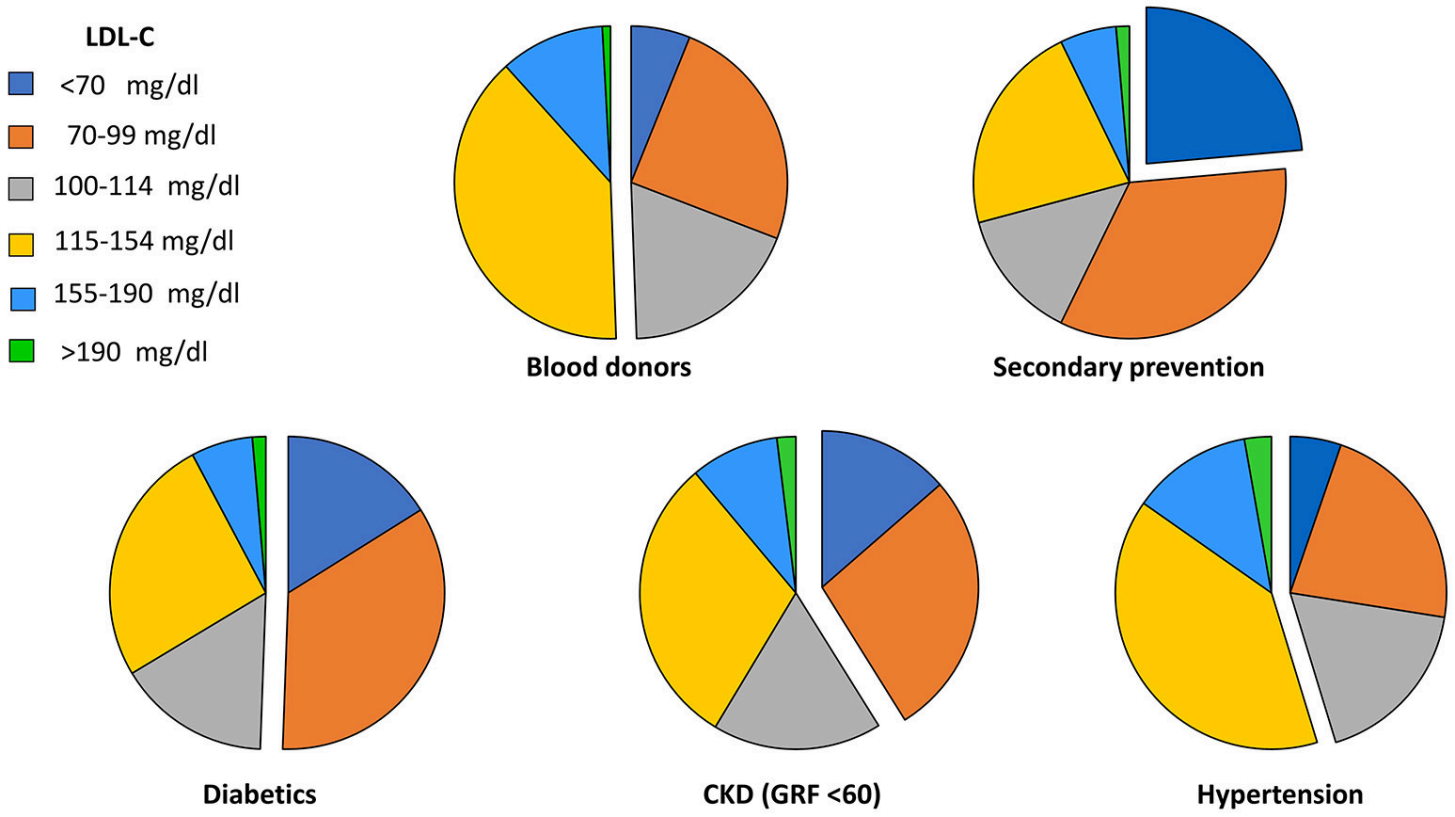
Proportion of patients at LDL-C targets (detached segments) according to their risk category. GFR, glomerular filtration rate.

SKIM LEAN focuses on the identification of subjects at high or very high CV risk to assess whether their LDL-C levels comply with personalized targets. Figure 6 shows that only one fourth of secondary prevention patients achieved the recommended target of <70 mg/dl, while among patients at high CV risk, levels below 100 mg/dl were observed in 51% of diabetics, 41% of patients with CKD, and 27% of hypertensive subjects. Conversely, among healthy blood donors at low CV risk, one half had LDL-C levels above 115 mg/dl.

## Discussion

The importance of hypercholesterolemia as a contributor to CV morbidity and mortality has long been known. Strategies for effective CVD prevention should be three-tiered. A decrease in the average cholesterol levels in the general population through lifestyle interventions and regulatory actions on the composition of processed foods may have huge impacts. In routine clinical practice this activity is typically carried out at the primary care setting and implies counseling on healthy lifestyle choices, that should start from childhood and be reinforced throughout adulthood. The second key CVD prevention activity is the identification of subjects at a higher risk of CVD events for comprehensive and intensive risk factor management. Routine laboratory testing offers the opportunity to spot subtle abnormalities in lipid and glucose profiles or renal function that may substantially impact on individual risk and should never be overlooked. The third key preventive activity, reducing risk in people with established CVD through therapies aimed at decreasing lipid levels, requires sustained clinical engagement since strict targets may not be easily achieved.

Epidemiological studies and surveys continue to document insufficient LDL-C control at all levels and highlight pitfalls in current hyperlipidemia management strategies. Our preliminary results in the large unselected population examined at NIG, a reference metropolitan hospital, confirm these findings and support the potential value of an intervention that is scalable to other settings and geographic locations.

Barriers to effective lipid management in clinic include a lack of focus on the global CV risk concept, insufficient awareness of appropriate targets relative to the individual patient's risk, inadequate knowledge of the therapeutic tools, and lack of confidence in personal treatment skills (Kwok et al., 2016). Therapeutic inertia is particularly worrisome in patients with established CVD, since over half of them may require the use of high-efficacy cholesterol-lowering medications (Poli et al., 2011).

The current use of administrative databases to identify patients from administrative coding, ordered tests and drug prescription refill, leaves important knowledge gaps, since LDL-C levels may not be properly appraised. The systematic exploitation of existing health care data holds a great potential to improve collaborative care.

Specialist support in the transfer of knowledge and in setting-up personalized drug treatment for complex cases represent powerful levers to improve dyslipidemia management in primary care. A shared care pathway will also reinforce patients' trust in physicians' sense of control of their health problem.

The main strength of SKIM LEAN is the automated iterative patient selection routine based on EHR processing which will seamlessly bring to medical attention those patients who most benefit from strict control of cholesterol, and will consolidate interactions among hospital specialists and community health professionals to foster high-quality patient care.

## Author contributions

LC, RD and MC conceived the project. CP, RD and MC wrote the article. MP, AA, PM, GM, FP, GO, FO and MT contributed to collect the data and to conduct the project. All authors have edited and approved the article.

### Conflict of interest statement

The authors declare that the research was conducted in the absence of any commercial or financial relationships that could be construed as a potential conflict of interest.

## Footnotes

^1^http://www.who.int/mediacentre/factsheets/fs310/en/

^2^Use of medicines in Italy - The OSMED report 2014 AIFA Italian Medicines Agency http://www.agenziafarmaco.gov.it/it/content/luso-dei-farmaci-italia-rapporto-osmed-2014

^3^*www.cuore.iss.it*

^4^Milan Local Health Authority Health Services Programming Coordination Document 2015. https://www.ats-milano.it

^5^*http://www.garanteprivacy.it/web/guest/home/docweb/-/docweb-display/export/378607*)
